# The impact of sex-role reversal on the diversity of the major histocompatibility complex: Insights from the seahorse (*Hippocampus abdominalis*)

**DOI:** 10.1186/1471-2148-11-121

**Published:** 2011-05-10

**Authors:** Angela Bahr, Anthony B Wilson

**Affiliations:** 1 Institute of Evolutionary Biology and Environmental Studies, University of Zurich, Winterthurerstrasse 190, 8057 Zurich, Switzerland

## Abstract

**Background:**

Both natural and sexual selection are thought to influence genetic diversity, but the study of the relative importance of these two factors on ecologically-relevant traits has traditionally focused on species with conventional sex-roles, with male-male competition and female-based mate choice. With its high variability and significance in both immune function and olfactory-mediated mate choice, the major histocompatibility complex (MHC/MH) is an ideal system in which to evaluate the relative contributions of these two selective forces to genetic diversity. Intrasexual competition and mate choice are both reversed in sex-role reversed species, and sex-related differences in the detection and use of MH-odor cues are expected to influence the intensity of sexual selection in such species. The seahorse, *Hippocampus abdominalis*, has an exceptionally highly developed form of male parental care, with female-female competition and male mate choice.

**Results:**

Here, we demonstrate that the sex-role reversed seahorse has a single MH class II beta-chain gene and that the diversity of the seahorse MHIIβ locus and its pattern of variation are comparable to those detected in species with conventional sex roles. Despite the presence of only a single gene copy, intralocus MHIIβ allelic diversity in this species exceeds that observed in species with multiple copies of this locus. The MHIIβ locus of the seahorse exhibits a novel expression domain in the male brood pouch.

**Conclusions:**

The high variation found at the seahorse MHIIβ gene indicates that sex-role reversed species are capable of maintaining the high MHC diversity typical in most vertebrates.

Whether such species have evolved the capacity to use MH-odor cues during mate choice is presently being investigated using mate choice experiments. If this possibility can be rejected, such systems would offer an exceptional opportunity to study the effects of natural selection in isolation, providing powerful comparative models for understanding the relative importance of selective factors in shaping patterns of genetic variation.

## Background

The impact of natural and sexual selection on genetic diversity has been intensively studied in both natural and captive-bred populations [1], but the majority of our current knowledge in this area is derived from species with conventional sex roles, with choosy females and competitive males [2,3]. Sex-role reversed species, in which females compete for mating opportunities and males are choosy [4,5], offer exceptional opportunities to investigate central tenets of sexual selection theory and the importance of sexual selection in the maintenance of genetic diversity.

The hypervariable major histocompatibility complex (MHC/MH) has proven to be a powerful model in which to investigate the importance of natural and sexual selection in shaping genetic diversity [6-8]. The MHC is an essential part of the vertebrate adaptive immune system, and includes a suite of more than 200 genes involved in the destruction of infected cells and the antibody response [9]. There are two major antigen-presenting groups of MHC molecules, class I and class II genes, which differ in their function, structure and pattern of expression [9]. The peptide binding region (PBR) of MHC loci encodes a groove that permits the binding of specific antigens, and this region typically exhibits the highest sequence polymorphism within the gene [10].

The investigation of MHC genes in a diversity of vertebrates indicates that these loci are more diverse than any other gene family [9]. Natural selection on MHC is thought to be driven primarily by pathogens, leading to balancing selection that acts on the PBR of MHC genes [11]. Balancing selection operates through either negative frequency-dependent selection, in which the relative fitness of individual alleles is influenced by their frequency (reviewed in [6]), or via heterozygote advantage. The advantage of MHC heterozygosity lies in the potential increase of the number of different parasite-derived antigens that can be detected by a MHC-heterozygous individual's immune system [12]. MHC diversity can be further enhanced by selection on linked genes, due to genetic hitchhiking [13,14]. In addition to the importance of MHC genes as an integral part of the adaptive immune system, MHC-mediated odor cues have been shown to be important in mate choice, kin recognition and inbreeding avoidance [15-19]. Disassortative mating is widely believed to promote MHC diversity and to increase the proportion of heterozygote individuals in natural populations [15,20,21]. Sexual selection can thus directly contribute to MHC allelic diversity via disassortative mate choice [12].

Despite consistently high levels of variation, there are major differences in the genomic organization of MHC genes in different vertebrate groups. While these loci are physically linked in mammals, class I and II genes are unlinked in bony fishes (class Actinopterygii) [22,23]. Due to the lack of linkage of MHC genes in actinopterygians, Stet et al. [23] have suggested that major histocompatibility genes in these species are most accurately termed MH loci. The unlinked nature of MH genes may provide increased evolutionary flexibility and contribute to enhanced MH diversity in this group. MH gene diversity is highly variable in teleost fishes, and while some species have a single classical MH class II beta-chain gene (MHIIβ) (e.g. salmonids [24,25]), most species have multiple copies of this locus (e.g. sticklebacks: 4-6 copies [26], perch: >8 copies [27], cichlids: >10 copies [28]). This variation may be due, at least in part, to ancestral chromosome or genome duplications [29].

While previous studies on teleosts have shown that both natural and sexual selection structure MH allelic diversity in species with conventional female-based mate choice [16,30,31], no study to date has investigated MH variation in sex-role reversed species in which mating decisions are made by the male. Males and females often differ in their ability to detect odor cues [32,33], and sex differences in the production, processing and use of MH-mediated signals are expected to influence the relative efficiency of sexual selection in sex-role reversed and conventionally-mating species, potentially reducing the level of MH variation in species with reversed sex-roles.

The teleost family Syngnathidae (seahorses and pipefish) is a well-suited model system to study questions concerning the relationship between sex roles and MH diversity. Both conventional and sex-role reversed species exist in the family and sex-role reversal has evolved several times independently in this group [34]. Studies of wild populations of the potbellied seahorse, *Hippocampus abdominalis*, have found evidence of female-female competition and male mate choice, suggesting that natural populations of this species are sex-role reversed [35].

Here, we characterize MH-variation in wild-caught and captive-bred individuals of sex-role reversed populations of the potbellied seahorse, a species with a highly developed form of male parental care. Genome sequencing and transcriptome screening confirm the existence of a single, highly variable copy of the MHIIβ locus in this species, with a pattern of variation identical to that detected in species with conventional sex roles. This pattern of genetic variation has been influenced by a combination of intralocus recombination and positive selection on sites believed to be important for peptide binding. MHIIβ is expressed in brood pouch tissues of male seahorses, suggesting that these molecules may be functionally active during male pregnancy. Our results indicate that sex-role reversed taxa such as the seahorse are capable of maintaining the high MHC diversity typical of vertebrate species with conventional sex roles.

## Results

### The seahorse, *Hippocampus abdominalis*, has a single MHIIβ locus

**Figure 1:**
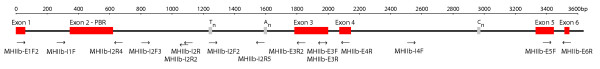
**Seahorse MHIIβ gene map**. The locations of exons, repetitive regions (A_n_, C_n _and T_n_) and primers used for genome walking and sequencing (see table 3) are indicated. The peptide binding region (PBR) of MHIIβ is located in exon 2. The full gene sequence has been submitted to GenBank (Accession #: HQ902181 and HQ902182).

Full-length gDNA sequencing of the seahorse MHIIβ locus from a single non-pregnant male identified 2 alleles, closely related to other teleost MHIIβ sequences (Blastn: *Hippocampus kuda*: e-value = 0.0, *Hippocampus *sp.: e-value = 2e-100, *Monopterus albus*: e-value = 2e-35, *Archoplites interruptus*: e-value = 1e-33, *Tetraodon nigroviridis*: e-value = 1e-33). The structure of MHIIβ in the seahorse is similar to that in other vertebrates, with 6 exons separated by 5 introns of varying length (Figure 1). The total intron length of the 2 full-length alleles differs, resulting in full gene sequences of 3508 bp and 3523 bp, respectively. Intron length variability is concentrated in 3 single-bp repetitive regions (A_n_, C_n _and T_n_) located in introns 2 and 4 (Figure 1).

Complete MHIIβ exon 2 sequences were obtained for 96 captive-bred and 5 wild-caught individuals. Irrespective of the primer combination used, a maximum of two alleles were found in all 101 individuals, indicating the existence of a single MHIIβ locus in this species. The comparison of parent-offspring MH profiles in 5 families of seahorses confirmed the Mendelian inheritance of the seahorse MHIIβ locus (Table 1). A 454-cDNA-library of the potbellied seahorse yielded 36 MHIIβ sequences (23 from pregnant pouch tissue (normalized/unnormalized: 18/5), 5 from non-pregnant pouch tissue (2/3), and 8 from normalized reference tissues) which could be assembled into a single contig identical to the MHIIβ genomic DNA sequence. cDNA sequencing indicated that the MHIIβ gene of the seahorse is expressed in muscle, liver and brood pouch tissue.

**Table 1:** Mendelian inheritance of MHIIβ in the seahorse

	Family A				Family B				Family C				Family D				Family E			
Individual	A1	A2	A3	A4	A1	A2	A3	A4	A1	A2	A3	A4	A1	A2	A3	A4	A1	A2	A3	A4
Father	3	4			2	6			5	13			4	9			2	6		
Mother			4	15			1	8			2	8			6	16			1	11

Juvenile 1		4	4			6	1			13	2		4		6			6		11
Juvenile 2	3			15		6		8	5		2		4			16	2			11
Juvenile 3		4		15		6	1		5		2			9		16		6	1	
Juvenile 4		4		15		6	1			13		8	4		6		2			11
Juvenile 5	3			15		6		8	5			8		9		16		6	1	
Juvenile 6	3			15	2		1			13		8		9	6		2		1	
Juvenile 7	3		4			6		8		13		8		9	6		2		1	
Juvenile 8		4	4			6		8		13		8	4			16	2			11
Juvenile 9	3			15	2		1		5			8								
Juvenile 10	3			15																
Juvenile 11	3		4																	
Juvenile 12	3			15																
Juvenile 13		4		15																

**Figure 2:**
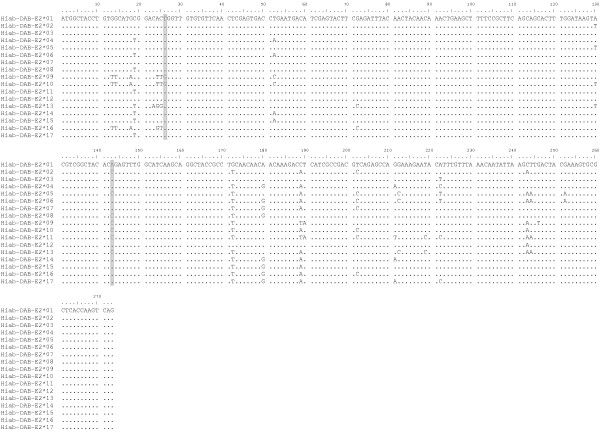
**Nucleotide alignment of exon 2**. 17 MHIIβ exon 2 sequences were obtained in 101 *H. abdominalis *specimens. Dots indicate identity to the first sequence. Synonymous substitutions are shaded in grey. Exon 2 sequences have been submitted to GenBank (Accession #: HQ902164 - HQ902180).

Combinations of MHIIβ alleles (A1-4) in parents and F1 offspring of 5 families of seahorses (A-E). See Figure 2 for allelic sequences.

### Sequence polymorphism in the PBR

**Figure 3:**
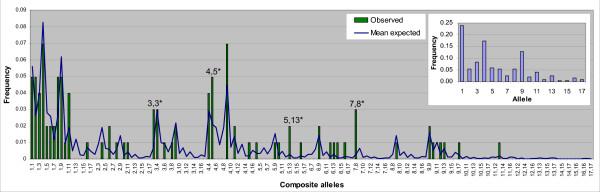
**Observed and expected allelic combinations**. Bars represent allelic associations observed in 101 *H. abdominalis *individuals. The mean expected frequencies of allelic combinations were simulated based on empirical allele frequencies (see figure inset). Asterisks indicate significant deviations (p < 0.05) from the mean expectation (N = 10,000 permutations). None of these outliers remain significant after a sequential Bonferroni correction for multiple comparisons.

**Figure 4:**
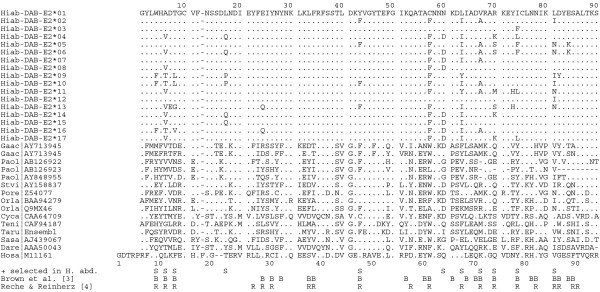
**Amino acid alignment of β1-domain**. MHII β1 sequences for *H. abdominalis *(Hiab), *Homo sapiens *(Hosa) and published teleost species (see methods). "S" represents positively selected sites in the seahorse as inferred from the exon 2 dataset, "B" indicates human PBS according to Brown et al. [10] and "R" reflects human PBS according to Reche and Reinherz [50]. Amino acid positions of the human MHII β1-domain are indicated below the human sequence.

**Figure 5:**
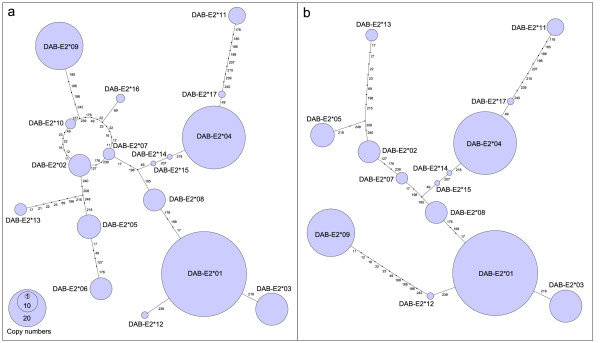
**Allele network of exon 2**. MHIIβ exon 2 nucleotide sequences for 101 *H. abdominalis *individuals. Circle sizes reflect allele frequencies. The positions of individual non-synonymous substitutions separating sequences are indicated. Figure 5a: All 17 alleles. Figure 5b: Recombinant alleles (RECCO: p < 0.05) have been removed (DAB-E2*06, DAB-E2*10 and DAB-E2*16).

Sequencing of the highly-variable peptide binding region of the seahorse MHIIβ locus identified a total of 17 *H. abdominalis *MHII β1-domain alleles in 101 individuals (Figure 2). 86% of individuals were heterozygous for MHIIβ (87 of 101), while 14% were homozygous, consistent with Hardy-Weinberg expectations (HWE Exact Test: p = 0.08). An analysis of allelic assortment detected 4 allele combinations more frequently than expected by chance (Figure 3; Hiab-DAB-E2*03/*03 p = 0.020, *04/*05 p = 0.040, *05/*13 p = 0.029, *07/*08 p = 0.001), but none of these values remained significant after correcting for multiple comparisons (Sequential Bonferroni correction). The 17 alleles include 25 polymorphic nucleotide sites and a total of 17 amino acid differences (Figure 4). Each of the 17 alleles differs by at least one amino acid substitution (Figure 4, 5). All alleles detected in wild individuals (Hiab-DAB-E2*01, 04, 05, 09, 13, 16 and *17*) were also detected in the captive-bred population. The nucleotide diversity π of the seahorse MHII β1-domain is 0.034. The dataset used for subsequent analyses contains 270 bp of exon 2 (total length: 273 bp), after omitting exon-spanning codons at the 5' and 3' ends of the exon (2 bp and 1 bp, respectively).

### A strong signal of positive selection

Only 2 of the 25 nucleotide substitutions detected in exon 2 of the seahorse are synonymous, leading to a dN/dS ratio of 3.7 (dN = 0.041, dS = 0.011, Table 2). A strong signal of positive selection was detected in this region (Z-Test p = 0.02), and 11 of the 17 variable amino acid sites are inferred to be under positive selection (p < 0.05, Figure 4) (seahorse sites 4, 6, 8, 17, 43, 60, 63, 67, 70, 74 and 81). A model incorporating positive selection fits the exon 2 dataset significantly better than a neutral model of evolution (M8 vs. M7, LRT = 46.744, df = 2, p < 0.01). Non-peptide binding sites in exon 2 show considerably less non-synonymous variation than do PBS (non-PBS dN = 0.018, PBS dN = 0.128) and exhibit no evidence of positive selection (dN/dS = 1.5; Z-Test p = 0.34; Table 2).

**Table 2:** Synonymous and non-synonymous substitution rates for exon 2 alleles of the seahorse MHIIβ gene.

Locus	Length (bp)	Samples	Alleles	dN	dS	dN/dS
Exon 2	270	101	17	0.041	0.011	3.73*
Exon 2, PBS	72	101	15	0.128	0.009	14.22**
Exon 2, non-PBS	198	101	9	0.018	0.012	1.50^ns^

Probabilities (*<0.05, **<0.001, ns = not significant) are derived from a Z-test (H1 = positive selection). Peptide binding sites (PBS) refer to the human sites, identified by crystallographic analysis in Brown et al. [10].

### Detection of recombination

An allele network based on non-synonymous substitutions was reconstructed to visualize relationships among the 17 unique MHIIβ alleles. The network shows no clear spatial structure, consistent with the pattern expected for a single locus (Figure 5a). The reticulative loop in the network suggests the presence of recombinant variants in the dataset, a hypothesis supported by statistical analyses (RECCO, p < 0.01), which indicate that 3 MHIIβ alleles are the result of intralocus recombination (Hiab-DAB-E2*06: p = 0.01, Hiab-DAB-E2*10: p = 0.01 and Hiab-DAB-E2*16: p = 0.03). A network without these recombinant alleles is qualitatively similar to the full network, but the placement of Hiab-DAB-E2*09 shifts in the pruned dataset, reflecting its high level of divergence from the central haplotypes (Figure 5b).

## Discussion

The sex-role reversed potbellied seahorse, *H. abdominalis*, has a single MHIIβ gene, which exhibits the typical vertebrate pattern of high genetic diversity. The existence of a maximum of 2 MHIIβ alleles per individual and the analysis of parent-offspring genotypes in 5 families of seahorses supports the Mendelian segregation of a single locus in this species. The high variability of the β1-domain of this gene, the region interacting with antigens, has been generated and maintained by a combination of positive selection and intralocus recombination, factors which have been shown to influence the pattern of MH variation in species with conventional sex roles [7,26]. The results of targeted gene sequencing are congruent with a transcriptome screen which indicates that a single copy of this locus is expressed in muscle, liver and brood pouch tissue of the seahorse. The expression of MHIIβ in pouch tissue of *H. abdominalis *males suggests that MH molecules may be immunologically active in brood-pouch tissues, and could possibly play a role in immune protection during the development of embryos in the paternal brood pouch [36].

### Genetic diversity

Previous studies of MHIIβ diversity in teleost fishes have demonstrated the exceptionally high diversity of this locus in this group (reviewed in [7]). These studies have, however, focused on species with conventional sex roles, with female-based mate choice and male-male competition (e.g. *Gasterosteus aculeatus *[37], *Oncorhynchus *spp., *Perca fluviatilis *[38] and *Poecilia reticulata *[39]). As males and females often differ in their ability to detect olfactory cues [32,33], the efficiency of odor-based MHC-mediated choice as a selective mechanism might be expected to differ between sex-role reversed and conventionally-mating species. Disassortative mating is thought to act together with pathogen-mediated selection to maintain MHC diversity [15,20], and species which lack the ability to detect and process MHC-based odor cues are thus expected to exhibit reduced levels of MHC diversity relative to species experiencing both forms of selection. Contrary to this hypothesis, MHIIβ diversity in the sex-role reversed seahorse is similar to that detected in other teleosts (see below), suggesting that sex-role reversed species are capable of maintaining the high MH diversity typical in other vertebrates. Both natural and sexual selection are thought to influence MH diversity [8], but the observation of high MHIIβ diversity in a sex-role reversed species suggests that natural selection may be sufficient to generate this high variability, a hypothesis which is currently being investigated using individual-based simulations (Ejsmond MJ, Radwan J and Wilson AB, in prep.). Alternatively, sex-role reversed species may indeed be capable of processing MH-based olfactory cues, something which is currently under investigation in targeted mate choice experiments in the seahorse.

MHIIβ gene-copy variation is high in teleosts, and while some teleost fishes have more than 10 functional copies of MHIIβ, a small number of species have only a single locus. Perhaps the best studied example of this are the ancestral tetraploid salmonids, who possess a single classical MHIIβ gene [25]. The high MHII β1-domain diversity of the potbellied seahorse is similar to that found in this group. The seahorse carries a similar number of alleles (*H. abdominalis*: 17 alleles in 101 individuals, *Oncorhynchus gilae gilae*: 5/142, *O. tshawytscha*: 12/144, *Salmo trutta*: 24/180, *O. mykiss*: 88/423), but exhibits fewer polymorphic sites (25 variable sites, 6.2% polymorphism) than that found in salmonids (21 - 70 variable sites, 7.7 - 27.2% polymorphism) [40-43]. *H. abdominalis *and salmonids show comparable nucleotide diversities in the PBR-containing β1-domain of exon 2 (*H. abdominalis*: π = 0.034; *O. gilae gilae*: π = 0.040 [43]; *S. trutta*: π = 0.054 [41]).

As interlocus gene conversion is thought to contribute to the diversity of gene families [44], one might expect to see higher intralocus variability in species carrying multiple MHIIβ loci. While species carrying several functional copies of MHIIβ possess a higher total number of alleles, intralocus measures of MHIIβ PBR diversity in these species are in fact less than those observed in species with only a single locus. Three-spined sticklebacks (*Gasterosteus aculeatus*), an important model system for the study of teleost MH evolution, are thought to carry at least 4 copies of MHIIβ [16,26,45]. A recent survey of 48 sticklebacks from locations in Europe and North America detected a total of 31 exon 2 alleles, or ≤ 8 alleles per locus [26]. Similarly, a survey of Trinidadian guppies, *Poecilia reticulata*, a species with at least 2 MHIIβ loci, recovered 18 exon 2 alleles in 56 individuals (alleles per locus ≤ 9) [21]. This pattern can also be observed in other species, for example in *Poecilia formosa *[46] and *Perca fluviatilis *[27], with 9 alleles in 29 individuals (≥ 2 MHIIβ loci; ≤ 5 alleles per locus) and 28 alleles in 58 individuals (≥ 8 MHIIβ loci; ≤ 4 alleles per locus), respectively. Methodological differences in the sample sizes and spatial scales of studies of MH variation complicate comparative analyses of genetic diversity, but the fact that species carrying a single MHIIβ locus have levels of allelic variation equal or greater than those detected in species with multiple copies of these loci (see above), suggests that intralocus allelic diversity of the MHIIβ PBR does not necessarily increase when more genes are present in a species. It is important to note, that maximal MHC diversity may also be constrained, both by interactions with the autoimmune response [47,48] and by consistently high levels of interlocus gene conversion, which may tend to homogenize genetic variation in species carrying multiple copies of these genes [49]. These factors may, in part, explain the lower than expected levels of MH variation detected in such species relative to species carrying a single copy of these genes.

### Peptide binding sites

We detected an excess of non-synonymous substitutions relative to synonymous substitutions in the PBR-encoding β1-domain of the seahorse, a pattern consistent with that found in species with conventional female-based mate choice. Due to the lack of X-ray crystallographic structure analyses of teleost MH genes, PBS in fishes are typically inferred by homology modeling to human MHC loci [50]. In addition, sites exhibiting a high variability and signatures of positive selection are also putative candidates for peptide binding sites [8,51,52]. 17 of the 90 MHII β1-domain sites of the seahorse are variable (19%), and 11 of these variable sites (65%) show evidence of positive selection. 9 of 11 sites correspond to human PBS as inferred by Reche and Reinherz [50] (Figure 4). While the length of the MHII β1-domain sequenced often differs between studies, several recent studies have analysed site-specific variation in the same 56 amino acid fragment of MHII β1, stretching from position 25 to 80 of the human alignment (Figure 4). A comparison among these studies indicates that the proportion of sites under positive selection in this region is similar between the sex-role reversed seahorse (6/56 = 11%), and conventionally mating salmonids (5-21%, [40]), *Poecilia *spp. (11-15%, [46]) and perch (22%, [27]), illustrating the striking consistency in the pattern of MH variation among species, despite differences in their sex roles.

## Conclusions

We provide the first data on the pattern of MH diversity in the seahorse (*H. abdominalis*), a species with an exceptionally well-developed form of paternal care and male mate choice. The sex-role reversed *H. abdominalis *exhibits levels of MHIIβ diversity similar to that detected in species with conventional sex roles. This species has a single functional MH class II beta-chain gene that is expressed in the male brood pouch, suggesting that this gene may be immunologically active in these tissues. The pattern of MHIIβ genetic diversity in the seahorse has been influenced by positive selection and recombination, and intralocus genetic diversity in this species exceeds that present in species carrying multiple copies of this gene. Mating experiments are currently being used to determine whether MH-odor cues are used in mate choice decisions in *H. abdominalis*, data which should help to shed light on the relative roles of natural and sexual selection in generating the high levels of MHIIβ diversity found in the seahorse.

## Methods

### Full-length MHIIβ gene sequencing

Whole genomic DNA was extracted from muscle tissue of a single *H. abdominalis *individual using a standard proteinase K/phenol-chloroform protocol [53]. To characterize MHIIβ genes in the seahorse, we first designed primers in conserved regions of the gene. These regions were identified using an alignment of published sequences for 11 teleost species (*Danio rerio - *Dare [GenBank:AAA50043], *Salmo salar *- Sasa [GenBank:AJ439067], *Cyprinus carpio - *Cyca [GenBank:CAD89312, CAA64709], *Tetraodon nigroviridis *- Teni [GenBank:CAF94187], *Oryzias latipes - *Orla [GenBank:BAA94279, BAA94280], *Poecilia reticulata - *Pore [GenBank:Z54077], *Stizostedion vitreum *- Stvi [GenBank:AY158837], *Paralichthys olivaceus *- Paol [GenBank:AB126922, AB126923, AY848955], *Gasterosteus aculeatus *- Gaac [GenBank:AY713945], *Hippocampus kuda *- Hiku [GenBank:AY211533], *Takifugu rubripes *- Taru [Ensembl:ENSTRUP00000004737], *Oryzias latipes *- Orla [Ensembl:ENSORLG00000000025]). Sequences were aligned in BioEdit v.7.0.9.1 [54] and primers were designed using Primer3 v.0.4.0 [55]. Primers used for MHIIβ sequencing are provided in Table 3 and their locations on the seahorse MHIIβ gene are indicated in Figure 1.

**Table 3:** Primers used to amplify and sequence MHIIβ in *H. abdominalis*.

Name	Sequence 5'-3'	Location
MHIIb-E1F2	GCCTCCTTTTCCTCACCTTC	Exon 1
MHIIb-I1F	TTGCGACTACACATTCAGCA	Intron 1
MHIIb-I2F2	TTTTTTTATCCCTTAACACTTAGAATACAG	Intron 2
MHIIb-I2F3	CGGGTCAACGAGTTCTCAAC	Intron 2
MHIIb-I2R	ACCAATGATTGTTCGGGTGT	Intron 2
MHIIb-I2R2	TCGGGTGTGATAATGGTCTG	Intron 2
MHIIb-I2R4	GGCGGCTGATTATCATGTTT	Intron 2
MHIIb-I2R5	TTGCGCCAAGGACCGGTTTAATG	Intron 2
MHIIb-E3F	GACGGCGACTGGTACTATCA	Exon 3
MHIIb-E3R	TGATAGTACCAGTCGCCGTC	Exon 3
MHIIb-E3R2	TCTGCTTGGGGTAGAAGTCG	Exon 3
MHIIb-E4R	AAGGCTGGCGTGTTCCAC	Exon 4
MHIIb-I4F	CGGGGGTCTTAAATCCTGTT	Intron 4
MHIIb-E5F	CTTTCCCTGGGAGGCTTC	Exon 5
MHIIb-E6R	TGGGAACCAGAATGCGACC	Exon 6

To amplify MHIIβ, we used long-range PCR under the following conditions: 1× ThermoPol reaction buffer (NEB), 1.2 μM dNTPs, 0.9 μM of each primer, 1.5 U of a 1:20 Pfu DNA polymerase (Promega) and Taq DNA Polymerase (NEB) mixture and approx. 60 ng DNA per 30 μL reaction. PCR running conditions involved an initial denaturation at 92°C for 5 min, followed by 35 cycles of 92°C for 30 sec, 58°C for 30 sec and 68°C for 0.5 - 4 min (depending on product length), with a final extension at 68°C for 5 - 15 min.

As the initial primer set provided only a fragment of the MHIIβ locus, genome walking was used to complete the sequence using a protocol modified from the Universal GenomeWalker Kit (Clontech). One μg of high-quality genomic DNA was digested separately with 10 U of the enzymes EcoRV (NEB), PvuII (NEB), StuI (NEB), DraI (NEB), AluI (Promega), HincII (NEB) and Cac8I (NEB) according to the manufacturer's recommendations. Purification of digested DNA and adaptor ligation followed the Clontech protocol. Genome walking was performed using a nested PCR approach with 1× ThermoPol reaction buffer, 1 μM dNTPs, 0.4 μM AP1 primer, 0.4 μM gene-specific primer 1, 1 U Taq DNA polymerase (NEB) and 1 μL of the DNA-adaptor-library in a 20 μL reaction volume for the first round PCR. The nested PCR was performed using the same protocol, but with the AP2 primer and a nested gene-specific primer along with 1 μL of a 1:50 dilution of the initial PCR product. Cycling conditions were identical in both PCRs, with 2 min at 92°C, 30 cycles of 30 sec at 92°C, 30 sec at 57°/60°/63°C and 3 min at 68°C.

PCR products were purified for sequencing using either a MultiScreen PCR filter plate (Millipore), gel-purification with the Wizard SV Gel and PCR Clean-Up System (Promega), or via cloning with a Topo TA Cloning Kit (Invitrogen) following the manufacturers' recommendations. 10-20 positive colonies per plate were picked into 25 μL of ddH20, directly PCR-amplified and sequenced. Cloned products were compared to direct sequences generated with several different primer combinations, in order to identify allelic phase and to identify any cloning-mediated PCR artifacts. Purified PCR products were prepared for sequencing by adding 1 μL Big Dye v3.1 Terminator Cycle Sequencing mixture (Applied Biosystems) and 1 μL primer to 2-8 μl of purified product in a 10 μL volume. Cycling conditions were 30 cycles of 10 sec at 96°C, 5 sec at 50°C and 4 min at 60°C. Ethanol-purified products were sequenced on an ABI 3730 automated sequencer (Applied Biosystems).

### Analysis of gene expression and MHIIβ copy number

To determine whether MHIIβ sequences obtained from genomic DNA represent functional alleles, we amplified and sequenced a partial MHIIβ cDNA sequence (exon 2 - 5) from liver, muscle and pouch tissue of a reproductively mature non-pregnant male seahorse. RNA was extracted using TRIZOL^® ^Reagent (Invitrogen) according to the manufacturers' recommendations. One μg of purified RNA was digested with 9 μL of DNase I (Promega) and reverse-transcribed into cDNA with 1 μL ImProm II Reverse Transcriptase (Promega) using 2 μL of a 500 μg/μL solution of a dT-adaptor primer (TAGGAATTCTCGAGCGGCCGCTTTTTTTTTTTT) in 25 μL volume. The program for the RT-PCR followed the manufacturer's recommendations (Promega). 3 μL of a 1:2 dilution of Millipore-purified cDNA was used as template in a PCR reaction with MHIIb-E1F2 and MHIIb-E6R under the standard PCR conditions outlined above.

Genomic DNA and cDNA sequencing indicate that *H. abdominalis *possesses a single functional MHIIβ gene (see below). To further explore this pattern, we screened cDNA libraries of seahorse pouch and reference tissues from pregnant and non-pregnant individuals for the presence of MH genes using 454 sequencing. Briefly, both normalized and unnormalized cDNA libraries prepared from purified total RNA derived from the pouch tissues of a single pregnant and non-pregnant seahorse, together with a pool of normalized reference tissues from the pregnant individual (brain, gills, liver, heart, kidney and testes), were individually MID-tagged with a unique sequence identifier. MID-tagged libraries were sequenced using GS FLX Titanium Chemistry (Roche), following the manufacturer's recommendations. A full plate of 454 sequencing yielded a total of 850 K filtered reads (average read length 230 bp), 92% of which could be assembled into 38 K contigs. The full results of this transcriptome screen will be described in detail elsewhere (Gauthier MEA, Stölting KN and Wilson AB, in prep.).

### Characterization of the MHIIβ peptide binding region (PBR)

In order to investigate the hypervariable PBR of MHIIβ, complete exon 2 sequences were amplified in an additional 100 individuals as part of a larger study investigating MH-based mate choice preferences in the seahorse. Seahorses are listed under Appendix II of the United Nations Convention on the International Trade in Endangered Species (CITES), and the majority of the samples included here thus originate from a captive-bred population derived from individuals collected from several sex-role reversed Tasmanian populations. The seahorses in this captive-bred population are held in large communal breeding tanks (2,100 L) with 50 males and 50 females per tank, allowing free mate choice (Hawkins R, pers. comm.). This population is genetically diverse (20 - 29 alleles per microsatellite locus; n = 4 loci) and an individual-based assignment test indicates the existence of a single Tasmanian population of captive-bred and wild-caught individuals (Structure: Pr(K = 1) = 1; see Additional file 1). A global test of microsatellite data failed to reject the null-hypothesis of Hardy-Weinberg equilibrium in this population (HWE Exact Test: p = 0.21). In addition to 95 individuals from the captive-bred population, we obtained complete exon 2 sequences from 5 wild-caught seahorses from Sydney, Australia (2 individuals collected in 2003) and Tasmania (3 individuals collected from 3 populations in 2003 and 2004). Genomic DNA from these individuals was extracted from fin clips using a DNeasy 96 Tissue Kit (QIAGEN). PCR products for exon 2 were generated using either primer MHIIb-E1F2 or MHIIb-I1F together with primer MHIIb-I2R4 (see PCR conditions above) and directly sequenced. Sequencing results were identical using either primer combination (data not shown). All private haplotypes were sequenced in a minimum of 2 independent runs in order to reduce the possibility of PCR artifacts. Degenerate positions in heterozygote sequences were scored using IUPAC nomenclature to facilitate the inference of allelic phase (see below).

### MHIIβ inheritance

We obtained exon 2 sequences from 47 F1 individuals from 5 families (n = 8-13 per family), to investigate whether MH alleles segregate in a Mendelian fashion. This approach demonstrates the mode of inheritance of these loci and provides a means to evaluate the reliability of sequence profiles generated for this fragment of the MHIIβ gene, through parent-offspring comparisons.

### Processing of sequences

Sequence data were assembled using Sequencing Analysis 5.2 (Applied Biosystems). Sequences were aligned with Muscle v.4.0 [56] and verified by eye in BioEdit v.7.0.9 [54]. To investigate the peptide binding region (PBR), we analysed 270 bp sequences of exon 2 (total length: 273 bp) after omitting the first 2 nucleotides and the final nucleotide of exon 2, to obtain a complete reading frame. As all exon 2 alleles are derived from a single MHIIβ locus (see below), they are named Hiab-DAB-E2*01-17, following standard terminology [57]. MH haplotypes of each individual were inferred from degenerate sequence data using a Bayesian statistical method implemented in PHASE v.2.1 using the default parameters [58], an approach which allows the determination of allelic phase from degenerate electrophoretic profiles [59]. SeqPHASE was used to convert between the PHASE input/output file and the sequence alignment [60].

### Analyses of sequence polymorphism

DnaSP v.4.90.1 [61] was used to calculate standard estimates of genetic diversity. To visualize relationships among the different exon 2 alleles and the non-synonymous substitutions separating them, a haplotype network was prepared using TCS v.1.21 [62]. The conversion of the sequence alignment file into a TCS-file was done with FaBox v.1.35 [63] and the final network was prepared using yED v.3.2.0.1 [64]. Tests for Hardy Weinberg equilibrium were performed in Genepop on the web [65,66] using the default settings for the Markov Chain search. The analysis of non-random associations of alleles was performed using non-parametric simulations (10,000 permutations), incorporating empirical allele frequencies, with the Monte Carlo simulation function in PopTools v.3.0.6 [67]. 95% confidence intervals of simulated data provided an estimate of expected frequencies of allelic combinations.

### Positive selection

dN and dS were calculated using Mega v.4.0.2 [68] under a Jukes-Cantor model. Mega v.4.0.2 [68] was also used to test for positive selection in the dataset, applying a Z-test under a Jukes-Cantor model (10,000 permutations). Site-specific positive selection was inferred using Codeml, implemented in the PAML v.4.2b package [69]. Codeml tests the goodness of fit of codon substitution models to a dataset using maximum likelihood. A neighbor-joining tree was generated for the 17 exon 2 alleles using Neighbor v.3.5c [70] under default settings, as implemented in BioEdit v.7.0.9. We compared the fit of a neutral evolution model with recombination (M7) with one allowing for positive selection (M8), using a likelihood-ratio test (LRT). Most previous studies on patterns of variation at vertebrate MHC loci have used the original human crystallographic structure of MHCIIβ prepared by Brown et al. [10] to infer putative peptide binding sites. More recently, Reche and Reinherz [50] presented an updated model of human PBS based on a larger sampling of potential peptides. In order to facilitate comparisons with previous studies, codons of the seahorse PBR were inferred through homology modeling to both of these datasets (see Figure 4). Given the more comprehensive dataset included in the Reche and Reinherz paper [50], PBS inferences in future studies should place greater emphasis on this work.

### Recombination

Recombination in the seahorse exon 2 dataset was tested using the default settings of RECCO v.0.93 (10,000 permutations) [71]. The identification of recombinant alleles with RECCO is based on a minimal cost solution, in which the relative cost of obtaining a sequence in an alignment from the other sequences by mutation and recombination is evaluated.

## Authors' contributions

AB participated in the design of the study, carried out the laboratory work and data analysis and wrote the manuscript. ABW conceived the study, supervised the laboratory work and data analysis and helped to draft the manuscript. Both authors read and approved the final manuscript.

## Supplementary Material

Additional file 1 **Figure S1: Genetic structure plot**. Click here for file
