# Supplementary material for: The impact of sex-role reversal on the diversity of the major histocompatibility complex: Insights from the seahorse (Hippocampus abdominalis)
Source: BMC Evol Biol. 2011 May 10;11:121. doi: 10.1186/1471-2148-11-121 (PMC3117728; doi:10.1186/1471-2148-11-121)

Additional file 1, Figure S1 – Genetic structure plot.

An individual-based analysis of genetic structure based on 4 neutral microsatellites best supports the existence of a single panmictic population (Pr[K=1] = 1.00) of aquaculture individuals (AQU) and samples collected from 3 Tasmanian localities (TAS1-3). The figure shows a structure plot for a two-population model (K = 2) with probabilities of individual assignment to the 2 hypothetical populations.

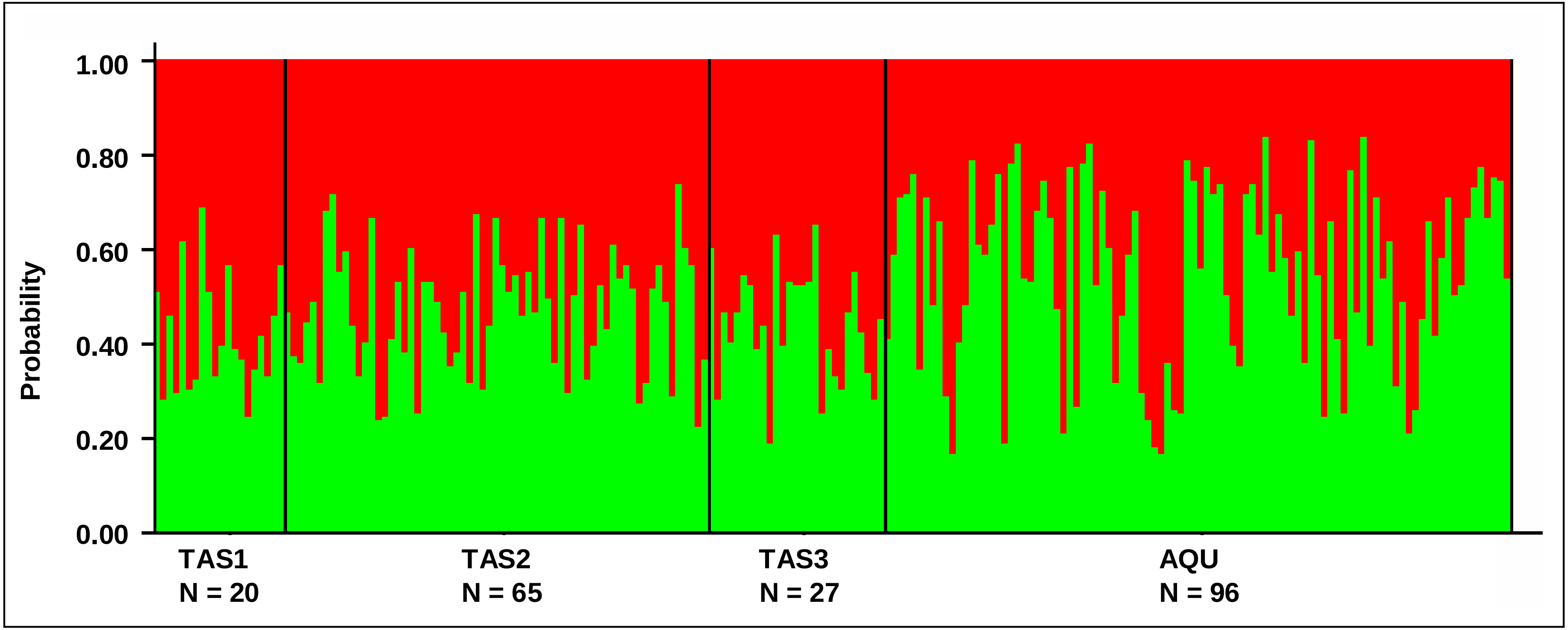

Supplement: Additional file 1 — Figure S1: Genetic structure plot. [file 1471-2148-11-121-S1.PDF]
